# Transcriptional Analysis of Tendril and Inflorescence Development in Grapevine (*Vitis vinifera* L.)

**DOI:** 10.1371/journal.pone.0092339

**Published:** 2014-03-17

**Authors:** José Díaz-Riquelme, José M. Martínez-Zapater, María J. Carmona

**Affiliations:** 1 Instituto de Ciencias de la Vid y del Vino (CSIC, Universidad de La Rioja, Gobierno de La Rioja), Logroño, España; 2 Departamento de Biotecnología, Escuela Técnica Superior Ingenieros Agrónomos, Universidad Politécnica de Madrid, Madrid, España; Instituto de Biología Molecular y Celular de Plantas, Spain

## Abstract

In grapevine (*Vitis vinifera* L.), the lateral meristem can give rise to either tendrils or inflorescences which are determined organs. To get insights into the processes of tendril and inflorescence development, we characterized the transcriptional variation taking place in both organs. The results of the global transcriptional analyses along tendril and inflorescence development suggested that these two homologous organs initially share a common transcriptional program related to cell proliferation and growth functions. In later developmental stages they showed organ specific gene expression programs related to the particular differentiation processes taking place in each organ. In this way, tendrils showed higher transcription of genes related to photosynthesis, hormone signaling and secondary metabolism than inflorescences, while inflorescences displayed higher transcriptional activity for genes encoding transcription factors, mainly those belonging to the MADS-box gene family. The expression profiles of selected transcription factors related with inflorescence and flower meristem identity and with flower organogenesis were generally conserved with respect to their homologs in model species. Regarding tendrils, it was interesting to find that genes related with reproductive development in other species were also recruited for grapevine tendril development. These results suggest a role for those genes in the regulation of basic cellular mechanisms common to both developmental processes.

## Introduction

Shoot development within the *Vitaceae* displays characteristic features that are rare exceptions in vascular plants [1]. Grapevine seedlings undergo a short-lived juvenile phase during which the shoot apical meristem (SAM) produce six to ten nodes bearing round leaves with a spiral phyllotaxis. Later on, phyllotaxis changes to alternate and leaf morphology becomes more lobulated marking the transition to the adult phase. In addition, the SAM starts to generate lateral meristems in a characteristic sequence. These lateral meristems, historically known as anlagen or uncommitted primordia [1], [2] generally give rise to tendrils. However, upon flowering induction, they differentiate inflorescences in place of tendrils [3], [4]. Based on their common origin, tendrils and inflorescences have long been considered as homologous organs [2], [5]. Furthermore, intermediate organs are frequently formed and tendrils and inflorescences can substitute each other depending on environmental conditions or hormonal treatments [3], [6], [7].

Consequently, flowering transition in grapevine does not seem to target the initiation of axillary meristems, as in other species, but the fate of those meristems, determining the developmental pattern of the modified shoots (tendrils or inflorescences) developing from them [3], [7]–[9]. In this way, under non inductive flowering conditions, lateral meristems follow a default developmental program to generate the climbing adapted shoots or tendrils. However, upon flowering inductive conditions, lateral meristems initiate a reproductive developmental program giving rise to inflorescences. In wild grapevine plants, flowering is induced once plants reach the forest canopy likely resulting from exposure to a rise in temperature and light intensity [3], [10]. Gibberellins and cytokinins have antagonistic effects in the control of flower initiation. Cytokinins promote the development of inflorescences from lateral meristem [3] while gibberellins (GAs), which promote lateral meristem initiation, inhibit their development as inflorescences and favor tendril development. In agreement with those observations, gibberellin insensitive grapevine plants bearing a dominant mutation at *VvGAI*, the Arabidopsis *GIBBERELLIN INSENSITIVE* (*GAI*) orthologous gene, are dwarfs with tendrils differentiating as inflorescences [6].

Grapevines grown in temperate regions generally require two consecutive growing seasons to complete their reproductive developmental cycle. Flowering is induced during the first season in latent summer buds in which the SAM produces 2–3 lateral meristems that become inflorescence meristems. Inflorescence meristems proliferate within the bud to give rise to inflorescence branch meristems with a spiral phyllotaxis and generate an immature raceme structure before the bud enters dormancy at the end of the summer. The next spring (second season), additional inflorescence branch meristems can be formed before each one gives rise to a cluster of 3–4 flower meristems that develop into flowers arranged in a dicasium [3], [8], [11], [12]. Flower development is initiated once the bud swells and shoot internodes begin to elongate. Flower meristems form sequentially sepal primordia, petals and stamens common primordia that soon divide to form separate primordia and finally the innermost carpel primordia [8]. Thus, the fate of the anlagen conditions a trait such as fertility (number of clusters per cane) which affects productivity.

We have previously characterized several grapevine genes possibly involved in the integration of flowering signals and the specification of inflorescence, flower meristems and flower organ identity [13]–[16]. These studies suggested a role for grapevine *FRUITFULL-LIKE* (*VFUL-L*) and *APETALA-1* (*VAP1*) in tendril development based on their unique expression patterns. Those results were the basis for a model to explain basic reproductive developmental processes in grapevine [9]. Given that plant reproductive development is mostly controlled at transcriptional level, we have now performed a transcriptional analysis of inflorescence and tendril development to identify both common and differential transcriptional regulatory patterns.

The results of this study suggest that tendrils and inflorescences, as homologous organs, share transcriptional components along their development mostly related to cell proliferation functions. However, they also show organ specific transcriptional patterns that can be related to their differential organ development and function. Interestingly some transcriptional regulators belonging to the MADS-box, the *SQUAMOSA PROMOTER-BINDING LIKE* (*SPL*) and the *FLOWERING LOCUS T/TERMINAL FLOWER 1* (*FT-TFL1*) gene-families, generally associated with reproductive development, seem to be also involved in tendril development.

## Results

### Transcriptome Variation along Tendril and Inflorescence Development

**Figure 1:**
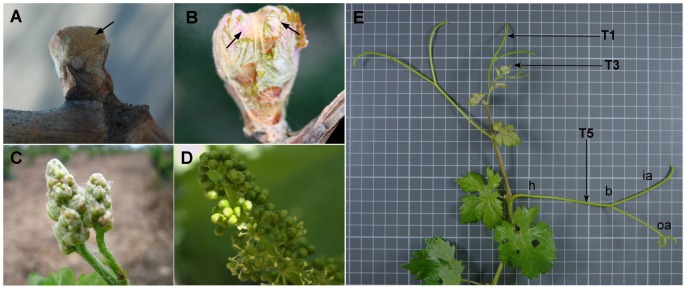
Developmental stages of inflorescences and tendrils. A: B2 stage bud from where inflorescence was excised; B: D stage bud containing two developing inflorescences; C: G stage inflorescence; D: I stage (25% bloom). E: Tendril stages; T1, T3 and T5 indicate respectively the first, third and fifth tendril from the apex. h: hypoclade; b: branching zone; ia: inner arm; oa: outer arm.

Development of grapevine inflorescences and tendrils, under our experimental conditions, was initially correlated with Baggiolini’s phenological stages [17] (Figure 1). At phenological stage B (Figure 1A), inflorescence branch meristems could still produce additional inflorescence meristems further differentiating into flower meristems. At stage D (Figure 1B), flower meristems had already been formed and sepal primordia were initiated in the outer region of flower meristems. The development of flower organs spanned phenological stages E to H. At stage G (Figure 1C), inflorescences were well developed, but flowers were not completely formed since differentiation of gynaecium is the latest and takes place along stage H. Finally, phenological stage I corresponded to the beginning of anthesis (Figure 1D). On the other hand, tendril development was initiated after bud break with the formation of an abaxial bract, closely followed by a sub-equal division of the tendril apex forming the inner and outer arm. As tendrils developed, both arms elongated and grew out to past the bract, reaching their final size (Figure 1E).

**Figure 2:**
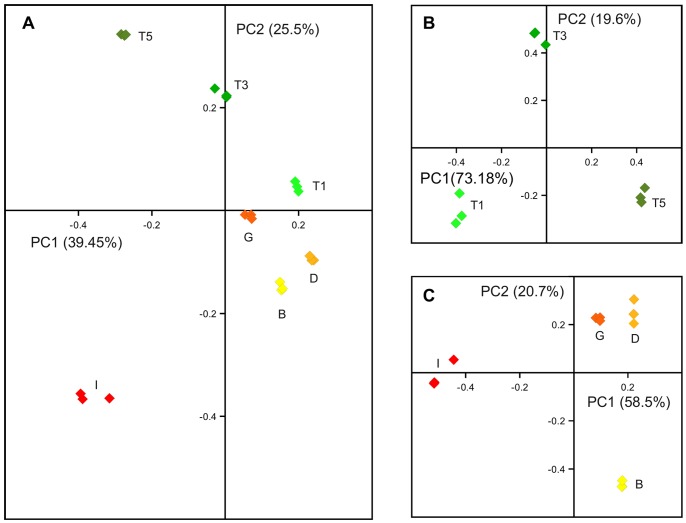
Bi-dimensional loading score plot of the sample replicates resulting from PCA analysis. A: whole experiment dataset; B: Tendril dataset; C: Inflorescence dataset. Percent of variation explained by each PC are shown in brackets. Replicate samples for the same time-point are in the same color.

In order to identify transcriptional changes related with the regulation of inflorescence and tendril growth and differentiation we perform a high throughput transcriptional analysis along inflorescence and tendril development using samples collected at four (inflorescence) or three (tendril) time points during the second growing season (see Methods). Principal Components Analysis (PCA) was performed on the whole expression dataset (Table S1) to confirm correlation among different biological replicates and to identify the main components of gene expression variation. As shown in Figure 2A, the results of the PCA plot showed consistency across biological replicates for every time point. The first two principal components explained 65% of the total variation in gene expression. PC1 could explain the time course evolution in both tendril and inflorescence development with young initial structures being placed to the right and mature structures to the left. PC2 distinguished tendril from inflorescences samples with T5 (tendril) and I (inflorescence) samples being the most divergent in the analyses. Furthermore, B, D and G inflorescence samples that contain stem tissue were closer to tendril samples than I samples only containing flowers (see Materials and Methods).

To further discriminate the main components explaining gene expression variation along tendril and inflorescence development, PCA was performed independently on each set of samples.

**Figure 3:**
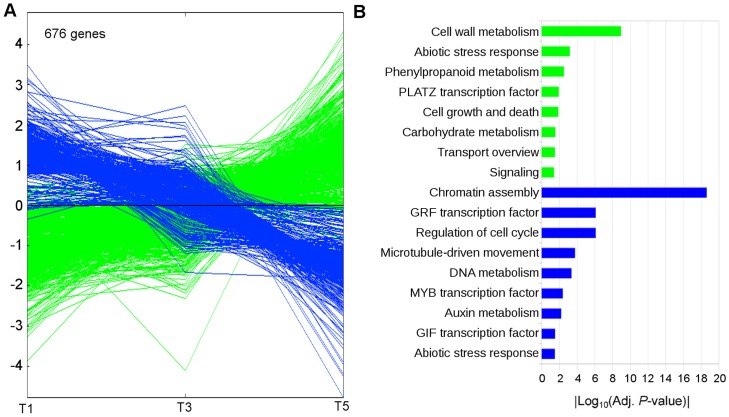
Expression profiles defining Principal Component 1 of transcriptome during tendril development. A: Expression profiles of the transcripts with positive or negative component score values higher than 3. Each single line represents the average of mean-centered expression values for an individual transcript. B: Functional categories over-represented in each cluster. Color code is the same as in A. Absolute values of the log_10_ transformed *P*-values were used for the bar diagram representing statistical signification, only categories with *P*-values <0.05 were shown.

For the tendril gene expression dataset (T1, T3 and T5 developmental stages, Table S1), PC1 explained more than 73% of gene expression variation apparently related to the time course of tendril development. By contrast, PC2 that differentiated T3 from T1 and T5 only explained 20% (Figure 2B). To investigate the biological basis of PC1, transcripts with the highest contribution to this component were identified according to their absolute component score (CS) value for PC1 (Table S1). Figure 3A shows the expression profiles of these transcripts that corresponded to 676 genes. Transcripts up-regulated **(**297 transcripts) are depicted in green. Transcripts down-regulated (379 transcripts) are depicted in blue (Figure 3A). Functional enrichment analyses indicated that the group of up-regulated transcripts was highly enriched in those encoding gene products involved in cell wall metabolism (specifically cellulose biosynthesis and pectin catabolism). Other categories were also significantly enriched, such as carbohydrate and phenylpropanoid metabolism (mainly lignin biosynthesis), cell growth and death (5 out of 8 genes putatively involved in cell death), signaling, transport and PLATZ (plant AT-rich sequence-and zinc-binding protein 1) family of transcription factors. On the other hand, down-regulated transcripts were highly enriched in those encoding gene products characteristic of actively proliferating cells (chromatin assembly, regulation of cell cycle, microtubule-driven movement, DNA metabolism and the GRF (GROWTH-REGULATING FACTOR family of transcription factors). Other categories significantly enriched were also related to cell proliferation such as auxin metabolism, GIF (GRF-INTERACTING FACTOR) and MYB families of transcription factors. Finally, enrichment of abiotic stress response functional category in both up and down-regulated transcript groups was mainly related to different stress responses, oxidative stress in the first group and drought stress in the second one.

**Figure 4:**
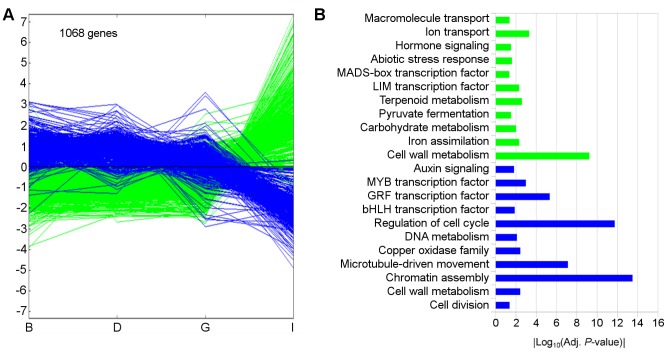
Expression profiles defining the Principal Component 1 for transcriptome of inflorescence development. A: Expression profiles of the transcripts with positive or negative component score values higher than 3. Each single line represents the average of mean-centered expression values for an individual transcript. B: Functional categories over-represented in each cluster. Color code is the same as in A. Absolute values of the log_10_ transformed *P*-values were used for the bar diagram representing statistical signification, only categories with *P*-values <0.05 were shown.

The same approach was applied to the analysis of gene expression changes during inflorescence development (Figures 2C and 4). In this case, PC1 was also related to the time course of inflorescence development and explained more than 58% of the expression variability, mainly distinguishing pre-anthesis stage from earlier inflorescence stages. As in the case of tendrils, up-regulated transcripts (563, green) were mainly enriched in those encoding gene products involved in the metabolism of cell wall (mostly pectin modification-related genes, such as polygalacturonases, pectate lyases, pectinesterase and expansin genes that are required for cell growth). Other significantly enriched categories identified during tendril development such as those related with carbohydrate metabolism, transport and abiotic stress response (related to oxidative stress responses), were also identified during inflorescence development. However, there were only a few shared genes in common categories enriched among up-regulated genes in both tendril and inflorescence development, suggesting the requirements of specific gene functions in the differentiation processes of these two organs (Table S2). In addition, other significantly enriched categories among up-regulated transcripts were specific of inflorescences such as hormone signaling, MADS-box (*VvBS1* and *2*, *VvAG1*, *VvAGL15.1* and *VvAGL66.1*), and LIM transcription factors. Similarly to tendrils, the most significantly enriched categories among the down-regulated transcripts (505 genes, blue) were those characteristic of actively proliferating cells (chromatin assembly, regulation of cell cycle, microtubule-driven movement, cell division and GRF transcription factors). The number of shared genes between tendril and inflorescences in these categories being high: 16 out of 19 in chromatin assembly, 13 out of 22 in the regulation of cell cycle, 6 out of 15 in microtubule movement, and 5 out of 5 in the GRF category. Other significantly enriched categories along both tendril and inflorescence development were those related with DNA metabolism and MYB transcription factors which also shared a high number of common transcripts in both organs.

### Differentially Expressed Genes along Inflorescence Development

**Figure 5:**
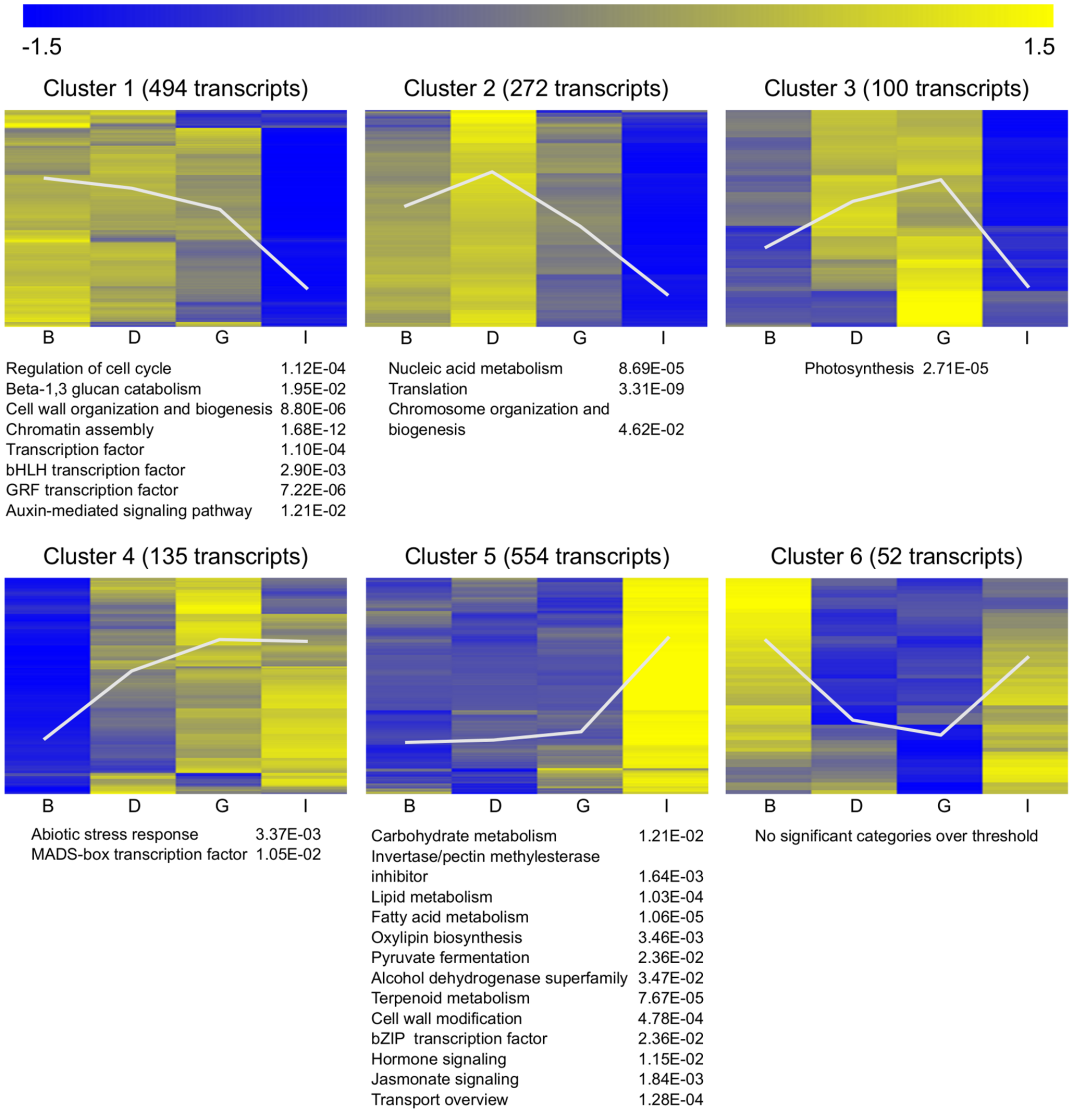
Hierarchical clustering of genes differentially expressed along inflorescence development. Significant genes (*P*-value <0.01) from differential expression analysis (ANOVA) were selected. Functional categories enriched in each cluster (*P*-value <0.05) are shown at the bottom. White lines represent the average expression pattern of the cluster.

The transcriptional complexity associated to inflorescence and flower development was further analyzed by differential expression analysis (ANOVA) and hierarchical clustering of expression values for the significant transcripts from stages B to I along inflorescence development. Cluster analyses identified six major clusters grouping up regulated and down regulated transcripts (Figure 5, Table S3). Cluster 1 grouped transcripts with the highest expression in B inflorescences and progressively decaying along development. These transcripts were significantly enriched in categories related to active cell proliferation (regulation of cell cycle, chromatin assembly, cell wall organization and biogenesis, auxin-mediated signaling and transcription factors belonging to bHLH and GRF families). Cluster 2 contained transcripts with highest expression in D inflorescences and abruptly decaying after this stage. This cluster was enriched in transcripts encoding products involved in nucleic acid metabolism, chromosome organization and biogenesis and translation, which are very active during the first steps of inflorescence and flower development. Most of them, together with those grouped in cluster 1, belonged to the same functional categories that were enriched among down-regulated transcripts contributing to inflorescence PC1 (Figure 4). The third cluster was enriched in transcripts corresponding to the photosynthesis category, which were up-regulated from B to G inflorescences and further decayed at stage I. This expression pattern reflects the transition from inflorescences into closed buds with no photosynthetic tissues (B stage) to emerging inflorescences (D stage) or young inflorescences (G stage). The drastic decay at stage I could result from the differences in samples tissue between G and I stages. I samples consisted of separated flowers and excluded inflorescence stems, probably with higher photosynthetic activity than the flowers. Cluster 4 grouped transcripts which expression increased significantly from B to G stages and maintained until I stage. This cluster was enriched in transcripts encoding products related to abiotic stress response and MADS-box transcription factors (corresponding to the *AP3*, *PI* and *AG* subfamilies), also contributing to PC1. Cluster 5 included transcripts with a very similar profile to those up-regulated in inflorescence PC1, although this analysis allowed identifying additional significantly enriched categories such as transport overview, fatty acid and lipid metabolism, jasmonate signaling and oxylipin biosynthesis, alcohol dehydrogenase superfamily, invertase pectin methylesterase inhibitor family and bZIP family of transcription factors. Finally, cluster 6 grouped transcripts with their maximal expression in B and I inflorescences but with no significant functional categories were enriched over threshold.

### Transcriptomic Differences between Inflorescence and Tendril Development

**Figure 6:**
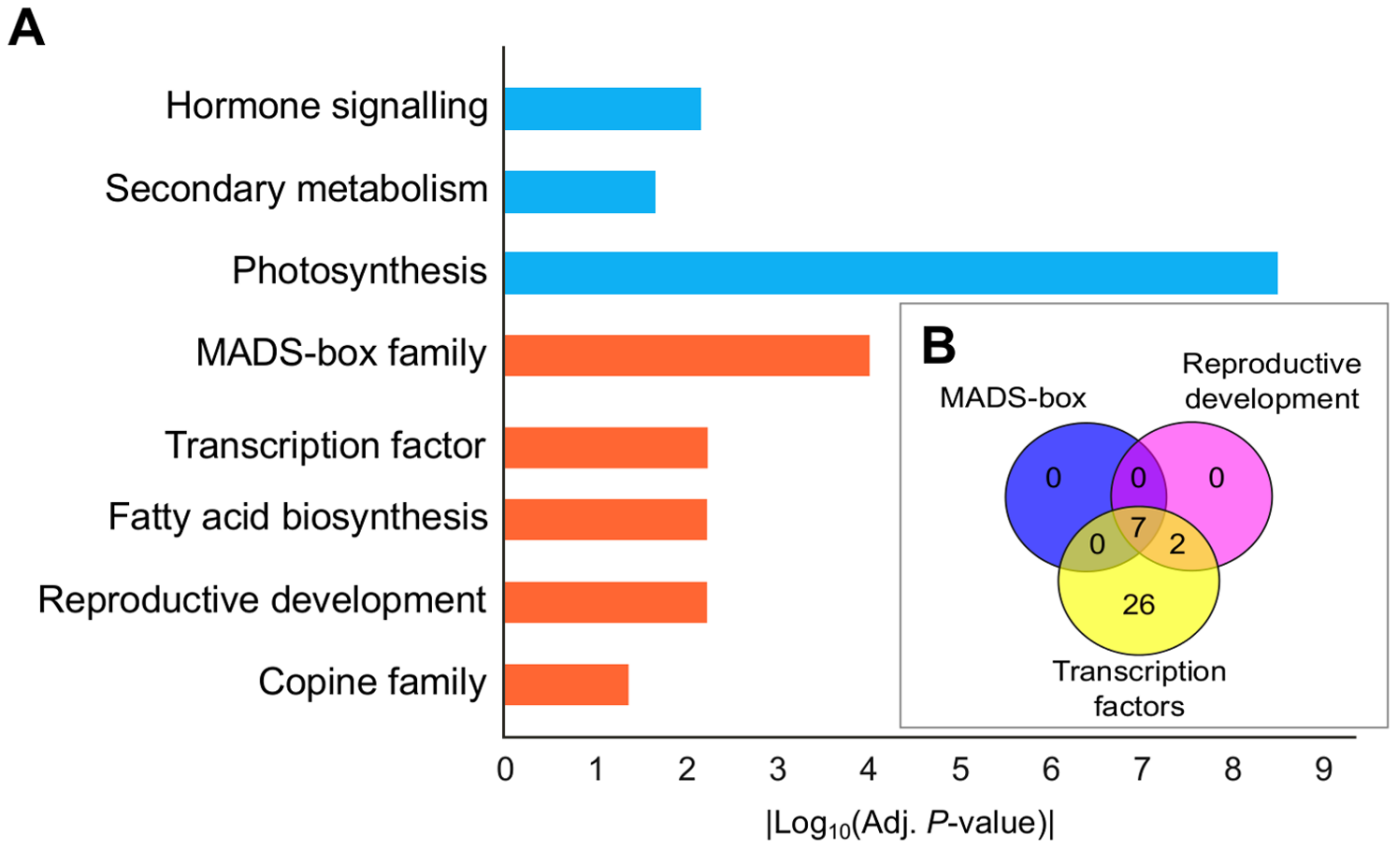
Functional categories over-represented in the tendril versus inflorescence comparison. A: Bar chart summarizes the significantly enriched functional categories between inflorescences and tendrils. Absolute values of the log_10_ transformed adjusted *P*-values (of the enrichment analysis) were used for plotting; only categories with adjusted *P*-values lower than the 0.05 threshold were shown; Bars are colored in blue for tendril categories and in orange for inflorescence ones. B: Venn diagram illustrating the overlapping enriched functional categories.

To identify transcriptional differences associated with specific organ development, differential expression between the earliest stages of tendril (T1 plus T3) and inflorescence (B plus D) development was analyzed. A T-test with a *P*-value threshold below 0.001 and a 2-fold expression cut-off identified 504 genes differentially expressed in early developmental stages of these two organs. Figure 6 summarizes the results of the functional category enrichment analysis from the differentially expressed transcripts between inflorescences and tendrils. These results showed that the major biological processes differentially active in tendrils versus inflorescences are photosynthesis, secondary metabolism (aromatic aminoacid metabolism, terpenoid biosynthesis, carotenoid and flavonoid biosynthesis) and hormone signaling (mainly auxin related signaling). This analysis also identified that the major biological processes differentially active in inflorescences versus tendrils corresponded to the transcription factor functional category as a whole suggesting a more complex regulatory network in the inflorescence than in the tendril. This category included the MADS-box family of transcription factors and the reproductive development category (Figure 6B). Transcription factor category included *VFL* transcript and an homologous of *Arabidopsis thaliana VERNALIZATION1* (*VRN1-4*) as well as members of the ABI3VP1, AP2, AS2, bHLH, DOF, YABBY, Homeobox, MYB, NAC, WRKY, G2-like and Zinc-Finger homeodomain-containing families. Transcripts belonging to the MADS-box family were those corresponding to B-function (*VvAP3.1 VvAP3.2* and *VvPI*) and E-function (*VvSEP 1, 2, 3* and *4*). Other significantly enriched categories were fatty acid biosynthesis as well as the copine family.

### Expression Profiles of Key Regulators of Reproductive Development

**Figure 7:**
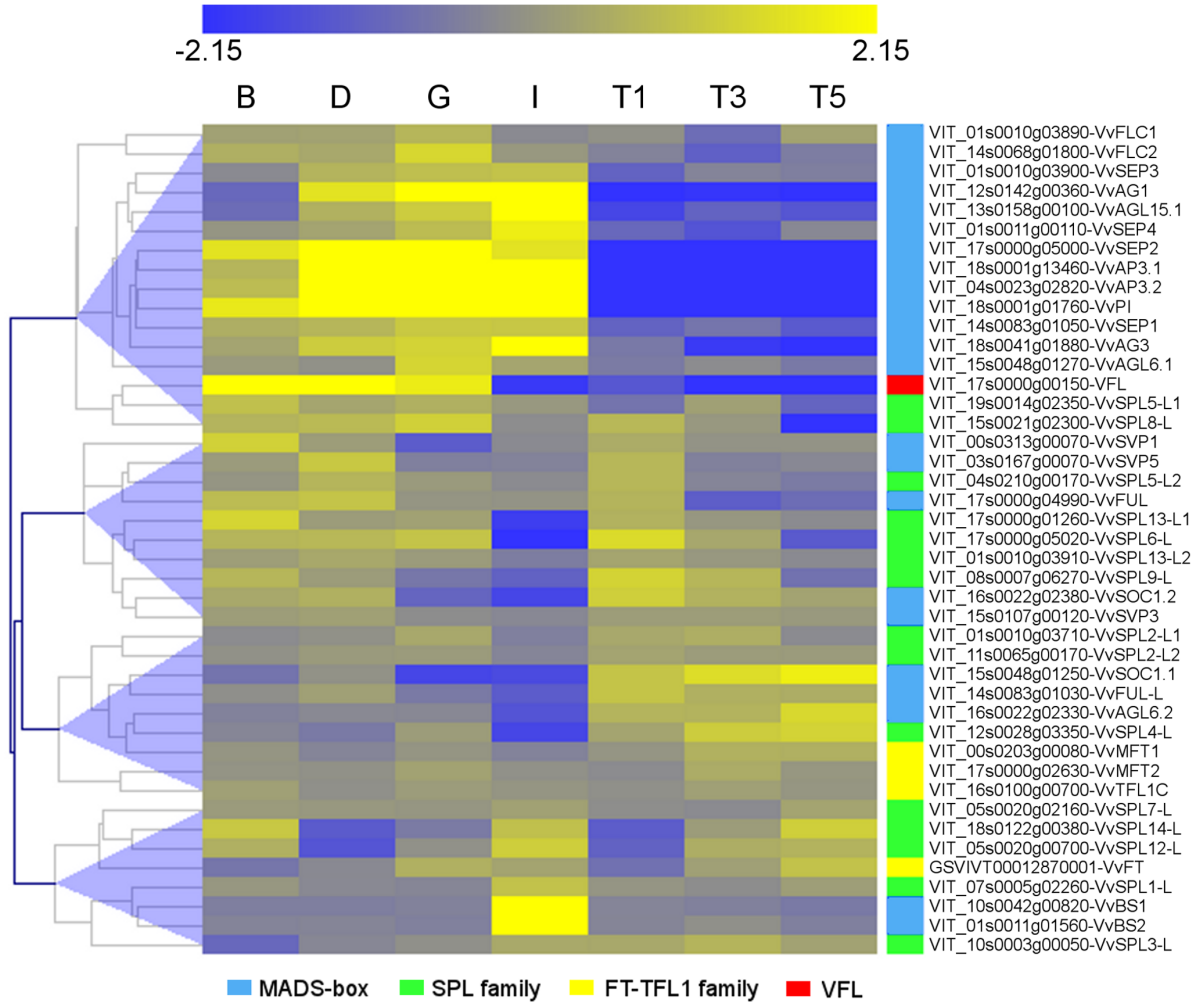
Expression profiles of key regulators of reproductive development along inflorescence and tendril development. Hierarchical clustering was performed using Pearson’s correlation. MADS-box gene family (blue boxes), *SPL* gene family (green boxes), *FT/TFL1* gene family (yellow boxes) and *VFL* gene (red box). Color scale (on top), represent mean-centered expression values.

Functional category enrichment provides a general view of the most active biological functions in a given developmental process. However, to identify putative genes involved in specific developmental processes, it is relevant to follow gene specific expression. This is particularly important for genes encoding transcriptional regulatory proteins. Therefore, we examined in detail the expression profiles of reproductive development regulatory genes such as *VFL* (the *FLORICAULA/LEAFY* ortholog in grapevine), the MIKC-type MADS-box genes, as well as the *SPL* and the *FT-TFL1* gene families. Hierarchical clustering based on expression values of these transcripts along tendril and inflorescence development are represented in Figure 7. Consistently with the major transcriptional profiles described in previous sections, expression analysis identified four distinct clusters. The first cluster grouped transcripts expressed along inflorescences but not in tendril development. It included transcripts likely associated with the events of flower meristem initiation and flower organs differentiation such as *VFL,* a clear representative of this cluster. Other transcripts in the cluster corresponded to B, C, D and E function MADS-box genes involved in the specification of flower organs identity as well as the homologs of *FLOWERING LOCUS C* (*VvFLC1, VvFLC2*) and *AGAMOUS-LIKE 15* (*VvAGL-15.1*). In addition, two *SPL*-like genes were also included in this cluster, *VvSPL5.1* and *VvSPL8-L,* given their preferential expression in inflorescences versus tendrils.

The second cluster contained transcripts with highest expression level in the first stages of both inflorescence and tendril development (B and D stage inflorescences and T1 tendrils). These transcripts belonged to the MADS-box and the *SPL* gene families. Three MADS-box genes belonging to the *SHORT VEGETATIVE PHASE* (*SVP*) subfamily were present in this cluster, *VvSVP1* and *5* were expressed in B and D inflorescences and in T1 tendrils while *VvSVP3* was detected at lower level in both organs. *VvFUL* that belong to the *AP1/FUL* subfamily of MADS-box genes was also preferentially expressed in B and D stages and in T1 as well as *VvSOC1.2* the putative Arabidopsis *AGL42* homolog. *SPL-L* genes in this cluster were *VvSPL5-L2*, *VvSPL6-L, VvSPL9-L*, and *VvSPL13-L1* and *2*.

The third cluster grouped transcripts mainly expressed in tendrils. Among them, there were three members of the MADS-box gene family (*VvSOC1*.*1*, *VvAGL6.2* and *VvFUL-L*), three *SPL* genes (*VvSPL2-L1* and *VvSPL2-L2* and *VvSPL4-L*) and also three members of the FT/TFL1 family (*VvMFT-1, VvMFT-2* and *VvTFLC1*).

The fourth cluster grouped transcripts with an opposite expression to those of cluster 2, with preferential expression at advanced stages of inflorescences (I stage) and tendril development (T3–T5). The cluster included two different expression groups. The first group contained three *SPL*-related transcripts (*VvSPL12-L*, *VvSPL14-L* and *VvSPL7-L*) mainly expressed in B and I inflorescence stages as well as T3 and T5 tendrils and the *VvFT* gene, that followed an expression pattern more intense in G stage inflorescences and in T5 tendril. The second group included transcripts for *VvBS1* and *VvBS2,* and *VvSPL1-L* which were restricted to inflorescence stage I, as well as *VvSPL3-L* that also showed tendril expression.

## Discussion

Grapevine tendrils and inflorescences are both determined lateral organs sharing a common ontogenetic origin. Their development include cell proliferation and cell differentiation phases that in inflorescences are extended after bud emergence and include flower meristem specification and flower organ differentiation. In fact, our transcriptional and functional enrichment analyses support the hypotheses that both organs share an initial phase of cell growth characterized by the expression of genes belonging to common functional categories. This phase is progressively switched off along development and substituted by a cell differentiation phase defined by transcript sets enriched in different functional categories in each organ.

### Common Transcriptional Changes Along Tendril and Inflorescences Development

Tendril and inflorescence development have some common features at transcriptome level, mainly related with basic processes of cell proliferation and organ growth. Both organs shared a large number of down regulated transcripts along their initial developmental phases. Most of these transcripts belonged to functional categories involved in cell growth and proliferation suggesting the existence of initial stages of rapid growth through cell division. Among the most significant down-regulated categories it is consistent to find the GRF and GIF transcription factors as well as auxin related metabolism and signaling protein encoding transcripts. GRF and GIF1 proteins form a functional complex involved in regulating cell proliferation via cell cycle control and determining the shape of lateral organs [18]. Genes of these families were expressed more strongly in immature organs and tissues than in mature ones in several species [18], [19]. GIF genes may affect the span of cell proliferation by modulating the expression level of cell cycle regulators and seem to be required in other developmental processes involving cell proliferation such as regulation of the plastochron and flower development [19]. Similarly, identification of functional categories related with auxin metabolism and signaling including transcripts with similar down regulation profiles suggest a relevant role of cell expansion in this initial proliferating phase that is progressively reduced [20]. The high number of common genes within the enriched functional categories among down regulated transcripts could suggest that they participate in basic aspects of cell proliferation.

In addition, tendrils and inflorescences shared the expression of key transcriptional regulators either at initial or late developmental stages. Inflorescence stages B and D and tendrils T1 shared the expression of genes belonging to the *SVP* subfamily of MADS-box and the *SPL* gene family (see Figure 7, cluster 2) that could be involved in the regulation of processes taking place in the first stages of determined organs development, such as cell proliferation. Expression of *SVP*-like genes in grapevine has also been observed in latent buds and in vegetative and reproductive organs such as roots, leaves, stems, flowers and fruits [15]. Similarly, in Arabidopsis *SVP* and *AGL24* transcripts were detected in many vegetative and reproductive organs [21]–[23].

Common *SPL-L* genes were expressed in early developmental stages of tendrils and inflorescences (*VvSPL5-L2*, *VvSPL6-L, VvSPL9-L*, *VvSPL13-L1* and *VvSPL13-L2*). Except the first one, they are all potential targets of grapevine miR156/7 (Figure S1) [24]. The *SPL* family of transcription factors is known to participate in the regulation of diverse plant developmental processes such as plant phase transition, flower and fruit development and plant architecture and could play similar roles also in grapevine [25], [26]. In addition, five *SPL*-related transcripts also showed expression in both tendril and inflorescences at later developmental stages (*VvSPL1-L*, *VvSPL3-L*, *VvSPL12-L*, *VvSPL14-L* and *VvSPL7-L*) These genes, except *VvSPL3-L,* do not show enough sequence complementarity with miR156 to be its potential targets. Some Arabidopsis counterparts of these genes (*SPL12*, *SPL14* and *SPL7*) also belong to the miR156/7 non-targeted *SPL* subfamily and are the largest proteins in the family [27]. Little is known about the functions of these putative transcriptional regulators with the exception of *SPL14*, which seems to regulate plant architecture and the length of vegetative phase, suggesting that this gene could play a role as a negative regulator of phase transition and flowering, having antagonistic function to other SPL proteins that promote vegetative phase change [28].

Another gene such *VvFT* was also detected in both tendrils and inflorescences but at later developmental stages (Figure 7, cluster 4). As previously described, *VvFT* expression in grapevine was associated to seasonal flowering induction in latent buds and to the development of inflorescences, flowers and fruits [14], similarly to what has been described for the *FT* gene in Arabidopsis [*29*]. We have previously shown that *VAP1*, the putative grapevine *AP1* ortholog, is expressed along tendril development [16]. Detection of *VvFT* expression in tendrils and inflorescences additionally supports the homology between those two organs. *AP1* was shown to be a downstream target of *FT* in Arabidopsis [30], [31] and the observed parallelism between the expression of *VvFT* and *VAP1* in grapevine could suggest the conservation of a similar regulatory network.

In conclusion, common transcripts seem to mostly represent genes that could be involved in basic developmental processes shared by both homologous organs as those related to cell proliferation and growth.

### Tendril Development Specific Transcriptome

Although most of the functional categories up-regulated in tendrils are also up-regulated in inflorescences, common genes in those enriched categories were scarce, suggesting the requirements of specific gene functions in the differentiation processes taking place in each structure. Up-regulated functions in tendrils are in concordance with the cell differentiation taking place during the development of this organ (Figure 3). These include cell wall metabolism, carbohydrate and phenylpropanoid metabolism, cell growth and death, abiotic stress response, signaling, transport and the PLATZ family of transcription factors, a class of plant-specific zinc-dependent DNA-binding protein. It has been suggested that this transcription factors could be involved in differentiation processes by negative regulation of cell proliferation [32]. Additionally, major functional categories identified among transcripts differentially expressed between both organs suggested a higher photosynthetic activity in tendrils than in inflorescences (Figure 6A) and revealed features related with the ability of tendrils to grow over supports (hormone signaling category) and also with the process of lignification taking place after tendril development and anchorage (secondary metabolism category).

It is noteworthy that the group of key regulators of reproductive development, mainly up-regulated in tendrils (third cluster, Figure 7) include three members of the MADS-box gene family (*VvSOC1*.*1*, *VvAGL6.2* and *VvFUL-L*). Among them, *VvSOC1.1* is the putative homolog of *SOC1* which plays a role as integrator of flowering signals from different pathways [33]–[35] and positive regulator of flower meristem identity genes such as *AP1* and *LEAFY* [35]. In grapevine, *VvSOC1.1* was one of the earliest MIKC-genes detected in latent buds which fit well with its putative role as flowering promoter [15]. In fact, it was surprising to detect expression of this gene along tendril development, showing the highest levels in fully developed T5 tendrils. Similarly, *VvAGL6.2* also showed its highest expression level during tendril development. *VvAGL6.2* belongs to the *AGL*-like clade that originated by a duplication of the *AGL6* subfamily in angiosperms [36]. This duplication resulted in two *AGL6* clades, eu*AGL6* that is predominantly detected in reproductive tissues and *AGL*-like that acquired expression in vegetative tissues and could be involved in developmental transitions of vegetative shoots. In agreement with the observed expression in other eudicots [36], *VvAGL6.1* showed the highest expression levels in stage G inflorescences (cluster 1, Figure 7A) whereas *VvAGL6.2* was tendril specific. Another MADS-box gene with significant expression along tendril development was *VvFUL-L*. This gene together with *VvAP1* are members of the *AP1/FUL* subfamily and have been previously shown to be highly expressed in tendril [15], [16]. In contrast, *VvFUL*, the third member of the subfamily, was mainly detected in latent buds and during flower meristem initiation and flower development. Notwithstanding, the three genes are expressed in latent buds during flowering transition which suggests a role in this process [15], [16] and is consistent with the proposed role for their Arabidopsis homologs (*AP1* and *FUL*) in the specification of inflorescence and flower meristem identity [37]. The involvement of members of the *AP1/FUL* subfamily in tomato leaf development [38] provides a broader perspective suggesting that this subfamily could participate in the control of cell proliferation and differentiation associated to lateral organ development. *SPL* genes in this cluster were *VvSPL2-L1*, *VvSPL2*-*L2* and *VvSPL4-L,* all of them are potentially targeted by miR156 in grapevine (Figure S1) [24]. Arabidopsis *SPL2* seems to be involved in lateral organ development within the reproductive phase [39]. In addition, three members of the *FT/TFL1* family are included in the same cluster (*VvMFT1, VvMFT2* and *VvTFLC1*). Altogether, the expression of genes generally involved in reproductive development along tendril development supports the hypothesis on the evolution of tendrils as climbing organs from initial reproductive organs. This evolution would have been conditioned by the functional divergence within these subfamilies and the novel roles acquired by genes recruited for tendril development. It is also possible that the biological function of these genes is not specifically related with inflorescence or tendril development but more generally involved in the regulation of lateral organ development either inflorescences, tendrils or other structures such as thorns or even leaves [38].

### Inflorescence and Flower Specific Transcriptome

Comparison of inflorescence and tendril transcriptomes showed a general enrichment in the transcription factor functional category in the inflorescence, suggesting a more complex regulation in this organ, consistently with its higher complexity. A number of these transcription factors are related to reproductive development (MADS-box family, *VFL* and *VRN1-4* transcripts). Most of the MADS-box genes were specifically expressed in inflorescence likely associated with the events of flower meristems and flower organs differentiation. Consistently, this cluster also included *VFL,* which Arabidopsis homolog *LEAFY* is required for flower induction and flower meristem specification. The different level of *VFL* expression in tendrils and inflorescences could suggest that a threshold level of *VFL* could be required for the development of inflorescence and flower meristem instead of tendril, as has been previously suggested [9]. Moreover, MADS-box genes involved in the specification of flower organs identity such as B function genes belonging to the *AP3/PI* subfamily (*VvPI*, *VvAP3.1* and *VvAP3.2*) [40]; C and D function genes (*VvAG1, VvAG2* and *VvAG3*) [41], [42]; and E function genes (*VvSEP1-4*) [15] were also detected following the expected expression pattern. Two other grapevine MADS-box genes showed expression in inflorescences, *VvFLC1* mainly in G stage and *VvFLC2* in B and G stages. *VvFLC1* and *VvFLC2* have been shown to be expressed during flowering induction in the first season and *VvFLC2* also during the dormancy period [43]. This pattern of expression is distinct from what has been described for Arabidopsis *FLC*, whose expression in the apex precedes the flowering transition and is also widely expressed in roots and leaves [44], [45]. Another MADS-box gene, *VvAGL-15.1*, was expressed at the highest levels in stage G and I. In Arabidopsis, *AGL15* is broadly expressed in vegetative and reproductive organs [46], [47] and in all tissues of embryos, declining in later stages of seed development and has been proposed to function as repressors of the floral transition, acting upstream of *FT* and probably in combination with other floral repressors like *SVP* or *FLC* [48]. Two *SPL*-like genes were also included in cluster 1, *VvSPL5.1* and *VvSPL8-L*. Both genes are also expressed during flowering induction in latent summer bud [43]. In Arabidopsis, *SPL5* belongs to the miR156/7-targeted *SPL* subfamily as in grapevine (Figure S1) [24] and act as a positive regulator of juvenile-to-adult phase change transition and flowering in Arabidopsis [49], [50], regulated by *SOC1* [34], while the miR156/7 non-targeted *SPL8* gene is involved in pollen sac development [51].

Moreover, cluster analysis along inflorescence development (Figure 5) allowed identifying additional significantly enriched categories. Enrichment in functional categories such as fatty acid and lipid metabolism, jasmonate signaling and oxylipin biosynthesis evidenced the importance of several related functions or processes in inflorescence development. Fatty acid biosynthesis is crucial in plant development, cell signaling and stress response acting as precursor of complex lipids or hormones biosynthesis such as jasmonic acid [52]. In Arabidopsis it has been shown a role of jasmonate in promoting anther and pollen development (synchronous pollen maturation, anther dehiscence, and flower opening) [53] thus suggesting a role for jasmonic acid in the last stages of flower development also in grapevine. In addition, functional categories such as alcohol dehydrogenase (ADH), pyruvate fermentation superfamily, invertase pectin methylesterase inhibitor family and bZIP family of transcription factors could also be specific of later stages of flower development. The invertase/pectin methylesterase inhibitor family plays important roles in developmental processes. In tobacco, invertase inhibitor NtCIF shows strong expression in the flower during later stages of flower development [54]. Finally, copine family also appeared as a significant category in inflorescences when comparing tendril versus inflorescence transcriptome (Figure 6). Copine proteins in Arabidopsis seem to be involved in promoting growth and development and in repression of cell death [55].

In summary, grapevine inflorescence and flower development showed extensive similarities with what has been described in Arabidopsis and other plant species, as evidenced also by previous results [13], [16], [41], [43]. Grapevine flower development seem to follow the ABCDE model first described in Arabidopsis, with the exception of *VvAP1*, which role in function A in grapevine has been questioned on the basis of its expression pattern [16]. Additionally, a threshold level of *VFL* expression seems to be crucial to specify the development of inflorescences and flower meristems instead of tendrils meristems.

## Materials and Methods

Grapevine (*Vitis vinifera* L. cultivar Tempranillo) samples were collected from an experimental vineyard at Finca El Encín, belonging to the Instituto Madrileño de Investigación y Desarrollo Rural, Agrario y Alimentario (IMIDRA, Alcalá de Henares, Madrid, Spain). This institution provided us access to grapevine experimental plots, whereas no formal permit was required because it is a public research institute. No protected species were sampled. Within the experimental vineyard we labeled three independent blocks of 240 plants each. Samples for each developmental stage were randomly collected from at least 10 plants per block.

Plant developmental stages were classified following the developmental series of Baggiolini (1952) [17] and the modified E-L system [56]. Inflorescence primordia from stage B were collected after hand dissection of early stage B buds from which inflorescences bearing only inflorescence branch meristems but not flower meristems were selected. Inflorescences D and G correspond to the first inflorescence of the shoot in phenological stages D and G respectively. Inflorescences I correspond to flowers of the middle part of inflorescences at stage I. Expression in tendrils was analyzed at three time points. Samples were collected from the first, third and fifth tendrils of stage I shoots. Tendril number 1 corresponds to the latest developed by the shoot apex and was processed as a whole. Samples of tendrils in third and fifth positions were taken from part of their three main regions: the inner and outer arms (a), the branching zone (b) and the hypoclade zone (h). Once collected, samples were frozen in liquid nitrogen.

### RNA Extraction

Total RNA was extracted from frozen samples according to Reid et al., 2006 [57]. RNA purification was performed using the RNeasy Mini Kit (QIAGEN) according to manufacturer’s protocols. To remove DNA traces in RNA samples, DNase I digestion was carried out with the RNase-Free DNase Set (QIAGEN). RNA integrity and quantity were assessed by Agilent’s Bioanalyzer 2100. Microarray hybridizations were performed at the Genomics Unit of the National Centre for Biotechnology (CNB-CSIC, Madrid).

### Microarray Data Processing and Analysis

Tendrils and inflorescences transcriptome were analyzed using Affymetrix GrapeGen GeneChip. Raw Affymetrix CEL files were imported to Robin software suite [58] to perform data normalization using the RMA method. Principal component analysis was performed to determine the major factors of expression variability using Acuity software (Molecular Devices, LLC, CA, US; http://www.moleculardevices.com/Products/Software/Acuity.html). The generated score matrix was used to select probe-sets that best fit the first principal component (PC1) selecting those scores greater than |3|. Likewise, probe-sets that best fitted PC2 were also chosen as those with component score greater than |3|.

Differential expression analyses were performed in MultiExperiment Viewer [59]. For differential expression between inflorescences and tendrils a T-test with a 0.001 cut-off for *P*-value and log_2_ ratio greater than |1| was used. For time-course analysis we used ANOVA with a 0.01 cut-off for *P*-value. *P* values were corrected using the Benjamini-Hochberg test.

To identify the biological functions over-represented within selected probe sets we performed functional enrichment analyses using FatiGO [60] (*P*-value <0.05). Functional categories were based on manual annotation of the custom made GrapeGen GeneChip, based on the 12Xv1 grape genome assembly, described in [61].

To represent the expression profiles of key regulators of reproductive development, genes were selected according to their functional annotation [61]. Expression values were extracted from the whole experiment normalized data matrix (averaged from the sample triplicates). When more than one probe set matched a single gene transcript, only the one that best BLAST matched was selected. Hierarchical clustering was performed using MultiExperiment Viewer [59] based on Pearson’s correlation and using the complete linkage option.

Gene annotation codes correspond to the V1 grapevine genome annotation and gene names were added from references in the literature for MADS box [15], *VFL* [13], *FT/TFL* [14] and in the case of *SPL* genes were developed in a previous work based on sequence homology with Arabidopsis genes [43]. These genes were selected from the reference *V. vinifera* annotation file hosted in VitisNet (http://www.sdstate.edu/ps/research/vitis/pathways.cfm) [62], the corresponding nucleotide sequences were obtained from CRIBI annotation tool (http://genomes.cribi.unipd.it/grape/get_annotation.php). Grapevine miR156 sequences were obtained from miRBase (http://www.mirbase.org/). Sequences were aligned using MUSCLE software [63] and those *SPL* genes with more than 90% identity with miR156 sequence were identified as potentially targeted.

Microarray data are available in the ArrayExpress database (www.ebi.ac.uk/arrayexpress) under accession number E-MTAB-2289.

## Supporting Information

Figure S1 **Sequence similarities between** ***VvSPL*** **genes and Vvi-miR156.** Heatmap summarizing percent identity between *VvSPL* genes and Vvi-mi156 resulting from the alignment of these sequences performed using MUSCLE.
(TIFF)

Table S1 **Whole expression dataset.** Dataset containing expression values for all the sample replicates. Also includes component score values (CS) for each probe-set in both tendrils and inflorescences series (for PC1, PC2 and PC3) and also in the whole experiment PCA (PC1, PC2 and PC3).
(TXT)

Table S2 **Gene-list from enrichment analyses of PC1 selected transcripts along inflorescence and tendril development.** File containing identifiers of common and differential transcripts either down-regulated or up-regulated in inflorescence or tendril datasets after selection by PC1 component score and subsequent functional enrichment analyses. Only those genes contained in categories with *P*-values <0.05 were shown.
(TXT)

Table S3 **Annotation of differentially expressed genes along inflorescence development.** File containing annotation information for all the significant genes (*P*-value <0.01) from differential expression analysis (ANOVA). It includes: unique identifier (Unique_ID), the name of the matching GrapeGen gene-chip probesets (Grapegen_probeset), the position in the 12X PN40024 genome assembly (Chromosome_position_12X), Cluster number regarding Figure 5 (Cluster), functional annotation of the specific transcript (Functional_annotation), VitisNet network(s) that contained the specific transcripts (Vitis-netNetwork), and the functional categorization (Functional_category) used for enrichment analyses.
(TXT)
