# Supplementary figures and images for: Transcriptional Analysis of Tendril and Inflorescence Development in Grapevine (Vitis vinifera L.)
Source: PLoS One. 2014 Mar 17;9(3):e92339. doi: 10.1371/journal.pone.0092339 (PMC3956920; doi:10.1371/journal.pone.0092339)

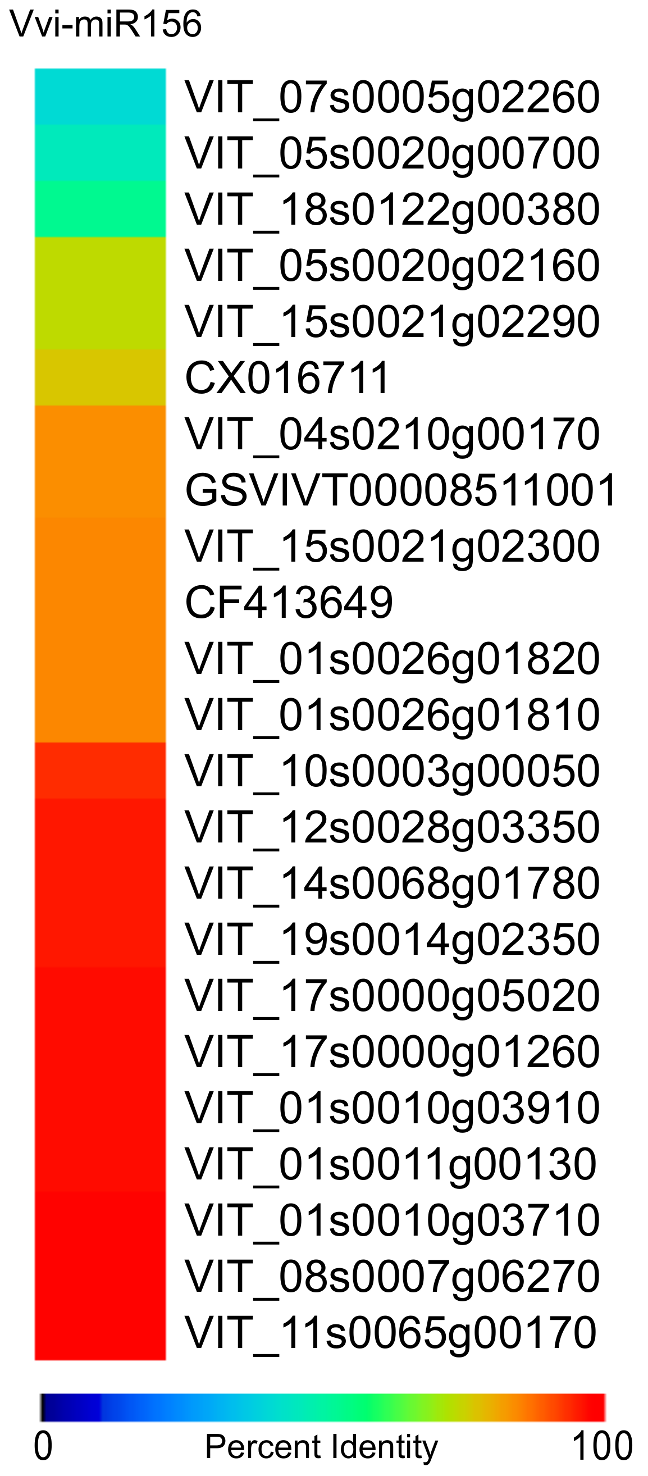

Supplement: Figure S1 — Sequence similarities between VvSPL genes and Vvi-miR156. Heatmap summarizing percent identity between VvSPL genes and Vvi-mi156 resulting from the alignment of these sequences performed using MUSCLE. (TIFF) [file pone.0092339.s001.tiff]
